# *Scedosporium apiospermum* infectious scleritis following posterior subtenon triamcinolone acetonide injection: a case report and literature review

**DOI:** 10.1186/s12886-018-0707-4

**Published:** 2018-02-13

**Authors:** Daisuke Todokoro, Junki Hoshino, Ayaka Yo, Koichi Makimura, Junko Hirato, Hideo Akiyama

**Affiliations:** 1 Department of Ophthalmology, Gunma University Graduate School of Medicine, 3-39-15 Showa-machi, Maebashi, Gunma 371-8511 Japan; 2 Laboratory of Space and Environment Medicine, Graduate School of Medicine, Teikyo University, Tokyo, Japan; 3 Clinical Department of Pathology, Gunma University Hospital, Maebashi, Japan

**Keywords:** *Scedosporium apiospermum*, Posterior subtenon triamcinolone acetonide injection, Infectious scleritis, Voriconazole

## Abstract

**Background:**

Ubiquitous fungi of the *Scedosporium apiospermum* species complex (SASC) cause various opportunistic infections. Posterior subtenon triamcinolone acetonide (STTA) injection is a standard therapy for intraocular inflammation and macular edema. We report a case of *Scedosporium apiospermum* infectious scleritis after a posterior STTA injection.

**Case presentation:**

A 75-year-old man received a posterior STTA injection to treat macular edema in his left eye. After 3 months, he complained of ocular pain and hyperemia in his left eye. Examination showed a subtenon abscess in the site corresponding with the STTA injection. After incising the abscess, the smear revealed numerous conidia-like structures. Although we suspected fungal infection and started topical voriconazole (VRCZ) and levofloxacin, the inflammation of the eye worsened. Fungal culture revealed filamentous fungus growth. Subsequently, we added systemic VRCZ and performed surgical debridement of the infected sclera and Tenon’s capsule. Pathology of the sclera showed fungal hyphae. The antifungal susceptibility test revealed low minimum inhibitory concentrations for micafungin, VRCZ and miconazole (0.06, 0.25 and 0.5 μg/mL, respectively). After 2 months, the ciliary injection subsided and VRCZ therapy was stopped. However, subtenon abscess recurred 1 month after discontinuation of topical VRCZ. Surgical debridement and topical VRCZ were resumed, with the eye finally improving after 5 months of management. The fungal species was identified as *Scedosporium apiospermum* sensu stricto morphologically and by DNA sequencing.

**Conclusions:**

This case was successfully treated by topical and systemic VRCZ and repeated surgical debridement. Infectious scleritis caused by SASC rarely develops after posterior STTA. SASC can produce conidia in the enclosed subtenon space. Late-onset infectious scleritis after a posterior STTA injection suggests the presence of a fungal infection, including SASC, thereby requiring extensive and prolonged medical and surgical treatment.

## Background

Infectious scleritis is a rare condition that can develop after ocular trauma, ocular surgeries that include pterygium excision with β-radiation or mitomycin C, posterior subtenon triamcinolone acetonide (STTA) injection, or it can be due to extensions from an adjacent ocular infection [1]. Triamcinolone acetonide belongs to the class of drugs referred to as long-acting corticosteroids. Posterior STTA injections are used as a standard therapy for treating intraocular inflammation and macular edema following various ocular diseases including uveitis, diabetic retinopathy and branch retinal vein occlusion [2–4]. The outside of the sclera is covered with the Tenon’s capsule, which consists of loose connective tissue and is located beneath the bulbar conjunctiva [5]. Posterior STTA injection is a technique used to directly introduce drug into the subtenon space. Adverse effects of STTA include scleral perforation, cataract formation, glaucoma, endophthalmitis, infectious scleritis (also called periocular infection) and blepharoptosis [6, 7]. Infectious scleritis following posterior STTA is a rare complication that is caused by bacteria or fungi [8–15]. Here, we describe a case of infectious scleritis caused by *Scedosporium apiospermum* sensu stricto, which was successfully treated by topical and systemic voriconazole and surgical debridement of the infected sclera.

## Case presentation

After a 75-year-old male, who pursued gardening as a hobby, noticed decreased vision in his left eye, he visited an ophthalmologist in September of 2015. He had a history of hypertension, colon cancer and metastatic hepatic tumor. As an ophthalmologic examination revealed macular edema in his left retina resulting from branch retinal vein occlusion (BRVO), he underwent a posterior STTA injection in his left eye to treat the macular edema. After routine disinfection of the lid margins and conjunctival sac with 10% and 0.625% (16-fold dilution) povidone iodine, respectively, the procedure was performed uneventfully. The patient once again visited his ophthalmologist in November due to complaints of ocular pain and hyperemia in his left eye. The symptoms did not improve even though he received oral and topical antibiotic treatment for 3 days. Computed tomography of the orbit was performed and revealed a peribulbar high density lesion of the left eyeball. Due to a suspected peribulbar infection, he was subsequently referred to our hospital in the middle of November.

**Fig. 1:**
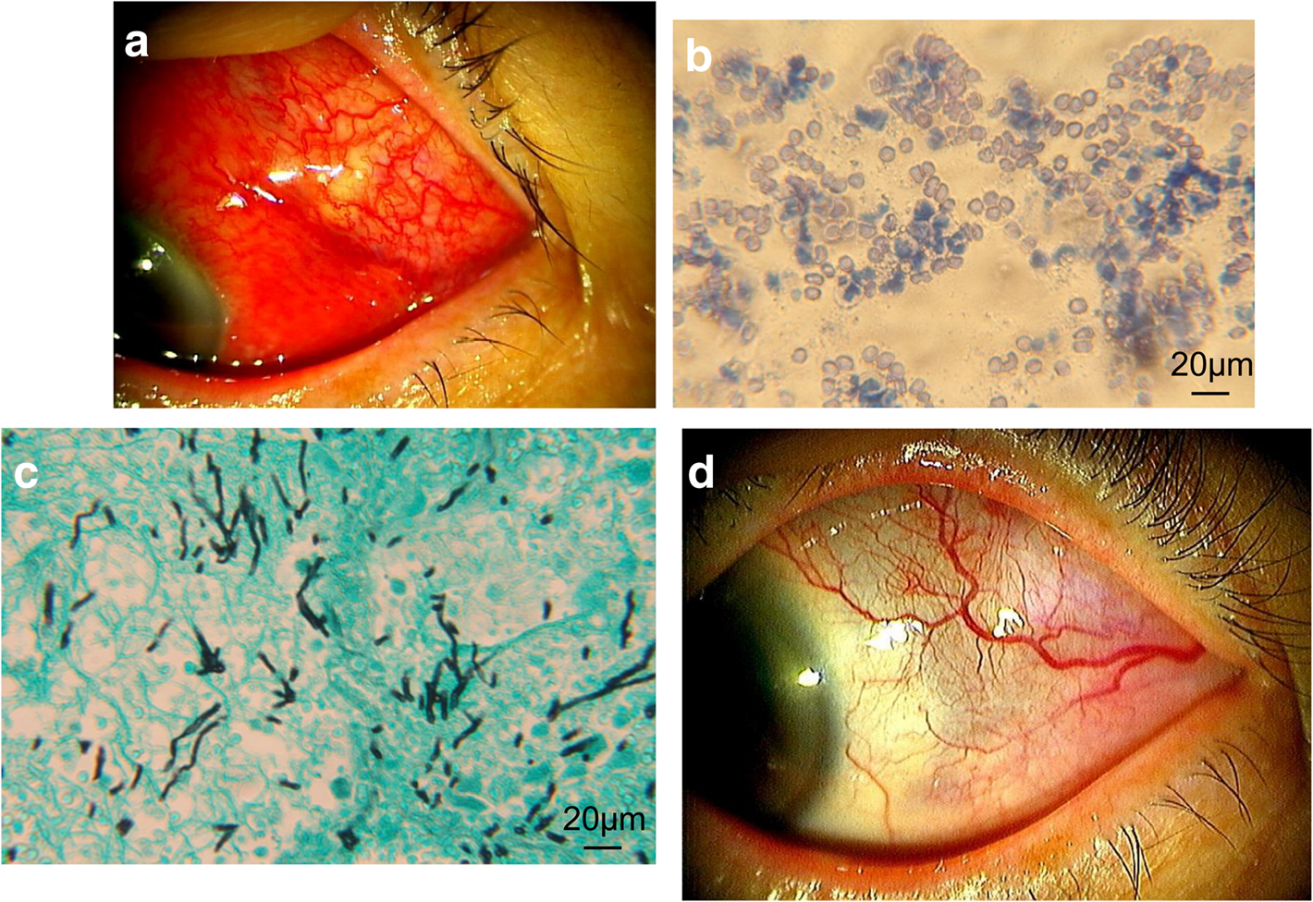
Slit lamp microscopy of the left eye with right down gazing at the first examination (**a**) shows ciliary injection and a subtenon abscess in the superotemporal quadrant where the posterior STTA injection had been previously performed. The content of the subtenon abscess smear was Gram-stained and observed by light microscopy (**b**). A large amount of round or oval conidia-like structures and polymorphonuclear leukocytes were observed. A scleral specimen obtained by surgical debridement was histologically examined using Grocott staining (**c**). Branching hyphae stained with black were observed. A slit lamp microscope of the same view at the final examination shows that the subtenon abscess and ciliary injection were absent (**d**)

**Fig. 2:**
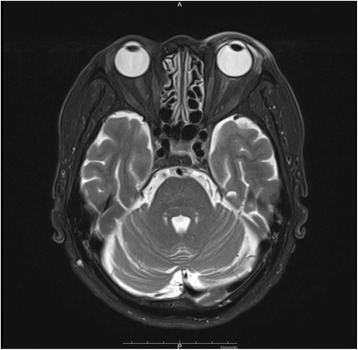
A horizontal section of the orbital magnetic resonance imaging shows a peribulbar high intensity lesion that corresponds to the subtenon abscess (T2-weighted image)

At his first visit, best corrected visual acuity (BCVA) (decimal) was 1.2 in the right eye and 0.15 in the left eye. Intraocular pressures were normal. Although the upper and lower eyelids of his left eye were swollen, we observed no restrictions of his eye movement. Slit lamp examination of the left eye showed ciliary injection, cells in the anterior chamber and a subtenon abscess in the superotemporal quadrant, which corresponded to the site of the posterior STTA (Fig. 1a). While our fundus examination did not reveal any intravitreal involvement, it did find macular edema and BRVO, which had been reported by his previous doctor. The right eye exhibited no abnormal findings, including both the anterior and posterior segments. The patient had no fever. The orbital magnetic resonance imaging (MRI) revealed the presence of a peribulbar high intensity lesion that corresponded to the subtenon abscess (Fig. 2). The MRI did not detect any paranasal sinus or intracranial invasion. The serologic test, which included white blood cells, C-reactive protein and beta-D-glucan, only revealed a slight elevation of C-reactive protein (1.11 mg/dL). To determine the causative pathogen, incision and drainage of the subtenon abscess were performed, with the obtained purulent fluid submitted for bacterial and fungal cultures. While the smears revealed polymorphonuclear leukocytes and a large number of round or oval conidia-like structures, a fungal filamentous appearance was not observed (Fig. 1b). Based on these results, we suspected fungal infectious scleritis induced by STTA. Therefore, the patient was initially started on 1% voriconazole (VRCZ) and 1.5% levofloxacin (LVFX) eye drops that were each administered six times per day in an outpatient department. Inflammation of the anterior chamber of the left eye worsened after 4 days, with choroidal detachment observed in the temporal region of the fundus. The fungal culture found an early colony of a filamentous fungus. Based on the definitive diagnosis of infectious scleritis by filamentous fungi, we then performed debridement of the infected sclera and Tenon’s capsule. The resected scleral tissue was submitted for further pathological examination. The conjunctival sac of the left eye was irrigated with 0.625% povidone iodine, followed by hospitalization for treatments with 1% natamycin ointment, 1% atropine eye drops and intravenous VRCZ. Ten days after the initial visit, we noted improvement in both his ocular pain and inflammation of the anterior chamber. As a result, we switched the voriconazole from an intravenous to an oral administration. Histopathological examination using Grocott staining revealed infiltration of branching hyphae into the sclera (Fig. 1c). Due to a novel subtenon abscess of the adjacent upper region of the left eyeball that developed at 18 days after the initial visit, we once again performed scleral debridement. The fungal species, *Scedosporium apiospermum*, was identified by DNA sequencing of the internal transcribed spacer (ITS) region of ribosomal DNA amplified by polymerase chain reaction (PCR) with ITS1F and ITS1R primers [16]. The strain was sent to Teikyo University for complete species identification. The susceptibility testing of the antifungal agents was performed using the broth microdilution method based on CLSI M38-A2 [17]. Results indicated low minimum inhibitory concentrations against micafungin, VRCZ and miconazole (Table 1). Based on the antifungal susceptibility test findings, the natamycin ointment was discontinued. Topical and systemic voriconazole administrations were continued for 2 and 1 months, respectively, and then stopped once the ciliary injection subsided. However, 1 month after the discontinuation of topical VRCZ, the subtenon abscess recurred at the same place (superotemporal quadrant). Incision and drainage of the abscess was performed three times. Light microscopic examination of the smears of the subtenon abscess revealed that septate hyphae were still present. As a result, we resumed the 1% VRCZ and 1.5% LVFX eye drops for 2 weeks and then stopped the administration. No recurrence was observed after the final treatment. Due to the prolonged macular edema, however, the final BCVA (decimal) of the left eye was 0.2 as of April 2016 (Fig. 1d).

**Table 1:** Susceptibility test of antifungal agents^a^

Antifungal agents	MIC^b^ (μg/mL)
Micafungin	0.06
Amphotericin B	16
Flucytosine	> 64
Fluconazole	32
Itraconazole	4
Voriconazole	0.25
Miconazole	0.5

^a^Broth microdilution method according to CLSI M38-A2

^b^Minimum inhibitory concentration

**Fig. 3:**
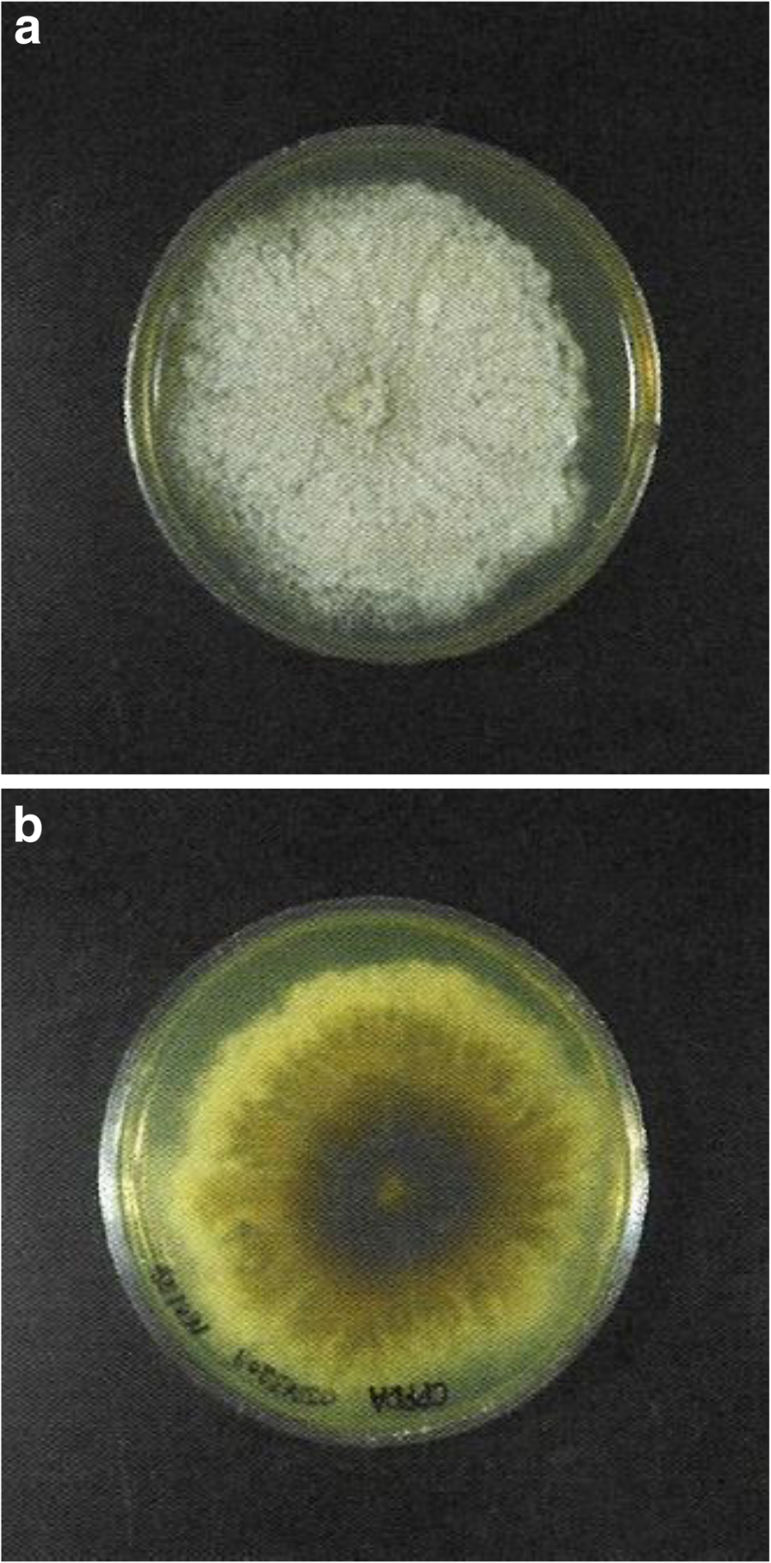
A *Scedosporium apiospermum* sensu stricto colony on potato dextrose agar incubated at 37 °C for 21 days shows white cottony appearance with an umbonate center on the surface (**a**). The reverse side shows a brownish dark wrinkle appearance at the center and a yellow-white appearance on the margin (**b**)

**Fig. 4:**
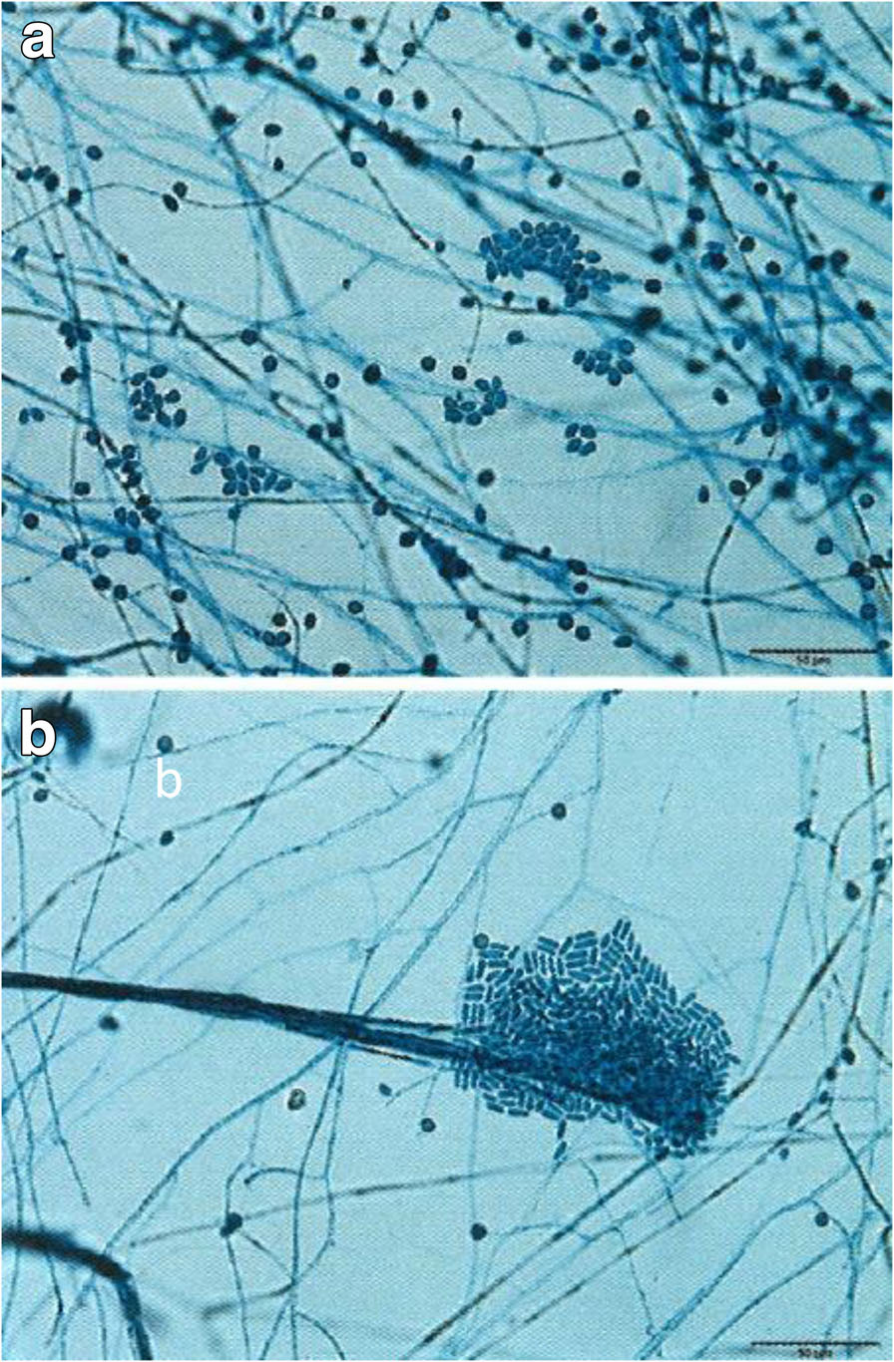
Microscopy of the slide culture on potato dextrose agar at 10 days revealed the presence of septate hyphae that were 2 μm in diameter. There was irregular branching of the hyphae with the production of round or oval (3 to 5 by 5 to 10 μm) lateral and terminal conidia (**a**). A *Graphium* synanamorph that produced a brush of cylindrical conidia (3 by 12 μm) was also observed (**b**)

Characteristics of the colonies found on the potato dextrose agar incubated at 28 °C for 21 days included a grayish white and cottony appearance with an umbonate center on the surface, and a reverse side showing a brownish dark wrinkle appearance in the center and a yellow-white appearance on the margin (Fig. 3). Microscopic features observed when using the slide culture technique on potato dextrose agar at 10 days included septate hyphae that were 2 μm in diameter and branching irregularly, along with the production of lateral and terminal conidia, which were round or oval (3 to 5 by 5 to 10 μm) (Fig. 4a). We also observed a *Graphium* synanamorph that produced a brush of cylindrical conidia (3 by 12 μm) (Fig. 4b).

In order to confirm the species identification using a molecular approach, the BUT locus of the β tubulin gene was also amplified by PCR using Bt1a/Bt1b primers [18, 19] and then applied to DNA sequencing [20, 21]. The obtained DNA sequencing was compared by using the Basic Local Alignment Search Tool. Both the ITS and the BUT exhibited 100% homology with that reported by Zouhair et al. for *Scedosporium apiospermum* (GenBank accession no. JQ690950 and JQ691056) [22]. Based on the current and previous results, we were finally able to identify the causative fungal strain as *Scedosporium apiospermum* sensu stricto.

## Discussion

Here, we report a rare case of *Scedosporium apiospermum* infectious scleritis that developed after a posterior STTA injection. The treatment of infectious scleritis caused by filamentous fungi is challenging due to the difficulty of species identification and performing an antifungal drug susceptibility test, the small number of available antifungal agents, the poor drug delivery to the deep sclera, and the persistence of filamentous fungi. Additionally, scleral infections can cause the development of endophthalmitis due to direct invasion into the eyeball, thereby requiring enucleation [23, 24]. The early detection of the causative pathogen, *Scedosporium apiospermum* during the early stages in the current case made it possible to successfully treat the patient and save his vision. After the antifungal susceptibility test revealed the low MIC of VRCZ, repeated scleral debridement was able to effectively remove large amounts of the fungi, thereby improving drug delivery to the deep scleral lesion. It should be noted that the polyenes, such as amphotericin B, have exhibited limited in vitro activity against most strains of *Scedosporium apiospermum* [25]. Though initial treatment by only topical VRCZ was ineffective, the inflammation improved after addition of intravenous VRCZ. Therefore, not only topical but both topical and systemic VRCZ proved to be effective in this case, which was consistent with the in vitro susceptibility results.

Posterior STTA injection is one of the standard options for treating uveitis and macular edema [2–4]. Infectious scleritis (periocular infection) is a rare complication that occurs after posterior STTA injection, with a reported incidence of 0.04% [6]. Table 2 lists the previous reports of infectious scleritis after posterior STTA injections. The causative pathogens of the scleritis include bacteria and filamentous fungi. Generally, bacterial scleritis develops within 1 month, while fungal scleritis develops after 2 to 3 months (Table 2). Since 4 out of the 9 reported cases exhibited recurrences after a transient improvement, this suggests that these types of infections require not only extensive but also prolonged systemic and topical medical treatment especially for fungal scleritis [26]. All of the gram-positive cocci cases recovered after antibiotic treatment and exhibited good visual outcome. However, all of the fungal cases required surgical treatment including debridement for persistent scleral infection and pars plana vitrectomy for endophthalmitis secondary to infectious scleritis. When encountering late-onset infectious scleritis that is suspected to be fungal, surgical debridement should be considered in order to make a definitive diagnosis. Interestingly, 3 out of the 4 reported fungal cases were caused by either *Scedosporium apiospermum* or *Pseudoallescheria boydii*, although unlike in our current case, the processes used for species identification were not detailed in the other reports. Thus, these results suggest that *Scedosporium apiospermum* species complex is the dominant fungi that cause infectious scleritis after posterior STTA injection.

**Table 2:** Reported cases of infectious scleritis after posterior subtenon triamcinolone acetonide injection

Patient	Reported year	Age and gender	Comorbidities (focal and systemic)	Durations from STTA	Symptoms at onset	Clinical ocular findings at onset	Causative pathogen	Pharmacotherapy (topical)	Pharmacotherapy (systemic)	Surgical therapy (except incision of abscess)	Recurrence	Outcome/final VA (decimal)	Reference
1	2004	90, female	BRVO	3 weeks	Orbital mass	Orbital mass, blepharoptosis	*Staphylococcus aureus*	None	Clindamycin	None		Recovery/NA	Engelman et al. [14]
2	2007	63, male	Graves’ ophthalmopathy	3 months	None	Subconjunctival abscess, anterior chamber cell, retinal white lesion and RD	*Alternaria* spp.	Fluconazole, miconazole, micafungin	Fluconazole, miconazole	Cataract extraction, PPV, scleral buckling	+	Recovery/0.7	Isshiki et al. [12]
3	2007	50, male	Uveoretinitis, Bechet’s disease, diabetes	2 weeks	Discharge	Subconjunctival whitish mass	*Nocardia* spp.	levofloxacin, cefmenoxime	Minocycline	None	+	Recovery/NA	Kusaka et al. [11]
4	2007	62, female	Diabetic retinopathy	2 month	Ocular pain	Periocular mass, conjunctival injection, anterior chamber cell	*Pseudallescheria boydii*	Amphotericin B, itraconazole	Amphotericin B, itraconazole	Debridement of subtenon abscess		Phthisis bulbi/NA	Oh et al. [10]
5	2007	33, female	Intermediate uveitis	2 weeks	Pain, redness and yellow discharge	Subconjunctival infiltrate	*Streptococcus pneumoniae*	ciprofloxacin	ciprofloxacin	None	+	Recovery/1.2	Azarbod et al. [13]
6	2008	80, female	CME after cataract surgery, POAG	A few weeks	Pain, red eye	Scleral necrosis, scleral thinning, nonrhegmatogenous RD	*Nocardia asteroids*	Amikacin, ceftazidime, trimethoprim sulfate, polymyxin B	Minocycline	Bovine endocardium patch graft		Recovery/CF	Seth et al. [9]
7	2009	58, male	Diabetic retinopathy	3 months	Diplopia, deep ocular pain	Ptosis, restricted eye movement, epibulbar abscess	*Scedosporium apiospermum*	Gatifloxacin, natamycin	Levofloxacin, itraconazole, voriconazole	PPV, cataract extraction		Recovery/0.5	Ikewaki et al. [8]
8	2011	20, male	Corneal graft rejection	2 days	Ocular pain, ocular discharge	Corneal edema, anterior chamber reaction, mucoid discharge, localized conjunctival necrosis	*Staphylococcus epidermidis*	Amikacin, vancomycin	Ciprofloxacin	None		Recovery/NA	Gharaee et al. [7]
9	2017	75, male	BRVO, hypertension, colon cancer and metastatic hepatic tumor	2 months	Ocular pain, hyperemia	Subtenon abscess, lid swelling, anterior chamber cells	*Scedosporium apiospermum* sensu stricto	Voriconazole, levofloxacin, natamycin	Voriconazole	Debridement of infected sclera	+	Recovery/0.2	This study

*STTA* subtenon triamcinolone acetonide, *VA* visual acuity, *BRVO* branch retinal vein occlusion, *NA* not applicable, *RD* retinal detachment, *PPV* pars plana vitrectomy, *CME* cystoid macular edema, *POAG* primary open angle glaucoma, CF counting fingers

*Scedosporium apiospermum* is one of hyaline filamentous fungi that are ubiquitously present in soil, sewage and polluted water [25]. While it was previously considered to be the asexual state of *Pseudallescheria boydii*, it has now been shown to be two distinct species [27–29]. Presently, *Scedosporium apiospermum* is considered to be a species complex that is comprised of *Scedosporium apiospermum* sensu stricto, *Scedosporium boydii*, *Scedosporium dehoogii*, *Scedosporium aurantiacum* and *Scedosporium minutisporum* [27]. The fungal isolate in the current study was definitively identified as *Scedosporium apiospermum* sensu stricto through the use of DNA sequencing of the β-tubulin gene, which is currently the most reliable identification method for this genus [29, 30]. *Scedosporium apiospermum* species complex (SASC) are important opportunistic pathogens [25], as they can grow at 40 °C in most species [29, 31]. These pathogens can cause mycetoma, traumatic arthritis, prosthetic joint infection, catheter-related infection, near-drowning syndrome, osteomyelitis, otitis externa, pneumonia particular to cystic fibrosis patients, and ocular infections including keratitis, scleritis and endophthalmitis [31–35].

The most remarkable point in our current case was the unique microscopic finding of the smear of the subtenon abscess. This subtenon abscess smear showed numerous conidia, with the pathology of the resected sclera revealing branching hyphae (Fig. 1b, c). These findings imply that this fungus was introduced into the subtenon space by a previous posterior STTA injection, with hyphae then growing into the patient’s sclera and producing conidia asexually in the enclosed subtenon space. This is the first report on the characteristics of the cytology and the pathology of a subtenon abscess associated with the SASC.

## Conclusions

We report a case of infectious scleritis that developed 3 months after a posterior STTA injection, with the isolates definitively identified as *Scedosporium apiospermum* sensu stricto. Surgical debridement combined with topical and systemic VRCZ administration effectively improved the infection and successfully saved the patient’s vision. This case report and the review of the literature imply that late-onset infectious scleritis after a posterior STTA injection strongly suggests the presence of a fungal infection, including SASC, thereby requiring extensive and prolonged medical and surgical treatment.

## Abbreviations

BCVA: Best corrected visual acuity
BRVO: Branch retinal vein occlusion
ITS: Internal transcribed spacer
LVFX: Levofloxacin
MIC: Minimum inhibitory concentration
MRI: Magnetic resonance imaging
PCR: Polymerase chain reaction
SASC: *Scedosporium apiospermum* species complex
STTA: Subtenon triamcinolone acetonide
VRCZ: Voriconazole
